# Microbial diversity in two traditional bacterial douchi from Gansu province in northwest China using Illumina sequencing

**DOI:** 10.1371/journal.pone.0194876

**Published:** 2018-03-23

**Authors:** Weibing Zhang, Qiaoqiao Luo, Yan Zhu, Jiang Ma, Lei Cao, Min Yang, Pencheng Wen, Zhongmin Zhang, Xiaoling He

**Affiliations:** 1 College of Food Science and Engineering, Gansu Agricultural University, Lanzhou, China; 2 College of Science, Gansu Agricultural University, Lanzhou, China; National Cheng Kung University, TAIWAN

## Abstract

Douchi has been consumed as a flavoring ingredient for centuries. During production of douchi, numerous microorganisms play important roles in the hydrolysis and conversion of proteins and starch, which are related to the quality and flavor of the end product. Therefore, in the present study, the microbial diversity in two types of home-made traditional bacterial douchi from Gansu province in northwest China was studied by high-throughput sequencing, and a corresponding analysis of the bacterial and fungal communities were conducted. The results showed that geography may have impacted the fungal diversity and the bacterial and fungal species richness in the samples. The results also showed that the microbial community was significantly different in samples of different origin and the difference in the microbial community at the genus level was greater than at phylum level. Two dominant bacterial genera (*Bacillus* and *Ignatzschineria*) were common to the two samples, both of which had a relative abundance of more than 1%. Four bacterial genera (*Staphylococcus*, *Aerococcus*, *Geobacillus*, and *Jeotgalicoccus*) were dominant only in the sample from Qingyang, while another four (*Carnobacterium*, *Proteus*, *Aneurinibacillus*, and *Enterococcus*) were dominant only in the sample from Longnan. Two dominant fungal genera (*Pichia* and *Candida*) were shared by the two samples. Additionally, two genera (*Rhodosporidium* and *Yarrowia*) were dominant only in samples from Longnan. The functional genes of the bacteria present in samples indicated that a significant difference was observed in the bacterial community between samples of different origin. We also found that microbial interactions between bacterial and fungal communities in the samples were very complex. This study provides previously unknown information regarding the impact of the environment on microbial communities in douchi and lays a foundation for further investigations into food ecology in bacterial douchi.

## Introduction

Douchi is a fermented food that is important for food flavor which has been produced in China and several Asian countries for thousands of years [1]. Besides its use as a condiment, it has been used to treat dyspepsia, restlessness, and asthma [2–3]. Recent studies have indicated that douchi can inhibit prostate and breast cancer [4]; it displays anti-diabetic activity, and protects against osteoporosis and cardiovascular diseases [5].

Douchi is normally made from soybeans by pretreatment and a two-step fermentation process (primary and secondary fermentations). Proteins and starch in soybeans are dissolved during the pretreatment process. During the fermentation process, a proportion of the proteins and starches are hydrolyzed and transformed into lactic acid, ethanol, amino acids and various aromatic components [6].Microorganisms have important effects on the quality and flavor of douchi, which are involved in hydrolysis and conversion of protein and starch [7]. Besides the starter, various types of microorganisms in different processing environments also participate in fermentation during the process of Douchi production [8]. Traditionally, microbial populations were characterized by culture-based techniques, which missed the vast majority of microbial diversity. These techniques are now replaced by modern culture-independent molecular techniques, such as denaturing gradient gel electrophoresis (DGGE) [9]. However, these techniques provide limited information regarding community structure because only a few sequences can be analyzed. High-throughput sequencing technology such as Illumina Mi Seq sequencing has been developed as a powerful tool to provide better assessment of microbial diversity because of its low cost, as well as its high read quantity and quality [10–14].

Douchi can be divided into four types according to the microorganisms predominantly involved in the fermentation, *Aspergillus*-type, *Mucor*-type, *Rhizopus*-type and Bacterial-type [7]. In the production of *Aspergillus*-type, *Mucor*-type, and *Rhizopus*-typedouchi, starters containing certain fungus are used to inoculate boiled soybeans for primary fermentation. However, in the production of bacterial-type douchi, no starter is used to inoculate the boiled beans, and naturally occurring microorganisms serve as the inoculum, instead. Therefore, the process of fermentation is largely affected by the local ‘house flora’, which may lead to significant differences in the microbial community between bacterial-type douchi and other types of douchi.

Numerous studies have been conducted to evaluate the microbial communities of fermented foods such as Plaisentif cheese, salami, and Pu-erh tea [15–18]. However, relatively little research has addressed the microbial community structure in traditional douchi without certain starters using modern culture-independent molecular techniques, especially Illumina Mi Seq sequencing. Qingyang douchi and Longnan douchi are two types of traditional home-made bacterial douchi without starter culture that have been produced in Gansu province for thousands of years. In this study, our objective was to generate an inventory of the diversity of microbial communities in Longna douchi and Qingyang douchi using Illumina Miseq approaches. The data generated, particularly differences in the distribution of particular taxonomic groups, were used to evaluate the effects of processing environments on the bacterial and fungal communities.

## Materials and methods

### Douchi samples collection

Douchi samples were collected in October, 2016. Samples from two kinds of home-made Douchi were purchased directly from local markets in different regions of China (Table 1).The time from production to sampling was within 10 days. All samples were placed into sterile tubes, numbered, and placed in a cooler for transportation to the laboratory for extraction of metagenomic DNA.

**Table 1:** Type of douchi and soybean, producers and origins of douchi samples.

Sample ID	Type of Douchi	Type of soybean	Producer	Origin
LN1	Bacterial type	Yellow	Gansu Longnan (home-made)	Gansu (North;33.33 N 105.58 E)
LN2	Bacterial type	Yellow	Gansu Longnan (home-made)	Gansu (North;33.33 N 105.58 E)
LN3	Bacterial type	Yellow	Gansu Longnan (home-made)	Gansu (North;33.33 N 105.58 E)
QY1	Bacterial type	Yellow	Gansu Qinyang (home-made)	Gansu (Sorth;36.03 N 107.88 E)
QY2	Bacterial type	Yellow	Gansu Qinyang (home-made)	Gansu (Sorth;36.03 N 107.88 E)
QY3	Bacterial type	Yellow	Gansu Qinyang (home-made)	Gansu (Sorth;36.03 N 107.88 E)

### DNA extraction

DNA was extracted from 0.2 g of the douchi samples using an E.Z.N.A.Soil DNA Kit D5625-01 (OMEGA, Norcross, GA, USA) according to the manufacturer’s instructions. The extracted DNA was quantified using a Qubit 2.0 spectrophotometer (Invitrogen, Carlsbad, CA, USA), and the integrity of the extracted DNA from the douchi samples was confirmed by electrophoresis in a 0.8% agarose gel.

### Illumina Mi Seq sequencing

Next generation sequencing library preparations and Illumina Mi Seq sequencing were conducted at Shanghai Personal Biotechnology Co., Ltd. (Shanghai, China).The bacterial 16S rRNA gene was amplified with the forward primer 520F (GCACCTAAYTGGGYDTAAAGNG) and reverse primer 802R (TACNVGGGTATCTAATCC) targeting the V4 region [19]. The fungal ITS rRNA gene was amplified with the forward primer ITS1-F (CTTGGTCATTTAGAGGAAGTAA) and reverse primer ITS2 (GCTGCGTTCTTCATCGATGC) targeting the ITS1-ITS2 region [20]. PCR was conducted using specific primers with barcodes and high-fidelity Trash Start Fastpfu DNA Polymerase (Trans Gen Biotech, China). The bacterial 16S rRNA gene PCR thermal cycle profile was as follows: 2 min at 98°C, followed by 25 cycles of 15 s at 98°C, 30 s at 55°C, and 30 s at 72°C and then final extension for 5 min at 72°C, after which the samples were held at 10°C. The fungal 18S r RNA gene PCR thermal cycle profile was similar to that of the bacterial profile except that it had five more cycles.

### Processing of high-throughput sequencing data

Amplicons were sequenced using a paired-end method by Illumina Miseq with a six cycle index read. Raw data generated from the high-throughput sequencing run were processed and analyzed following the pipelines of Mothur (V.1.31.2) and QIIME (V1.7.0) [21–22]. Sequence reads were trimmed so that the average Phred quality score for each read was above 20. After trimming, these reads were assembled using the Flash software (V.1.2.7) [23] and reads that could not be assembled were discarded. Chimera sequences were identified and removed using UCHIME (V.4.2) [24]. Quality sequences were subsequently assigned to samples according to their unique 7 bp barcode and sequence clustering was performed using the UCLUST algorithm with a similarity cutoff of 97%, after which samples were clustered into operational taxonomic units (OTUs) [23].

The taxonomic identities of bacterial OTU representative sequence were classified with the Ribosomal Database Project (RDP) classifier with the SILVA databases [25], while the fungal OTU sequences were classified by standalone Mega BLAST searches of the UNITE database [26].

This dataset was available in the SRA at the NCBI under accession number SRP128487 (https://www.ncbi.nlm.nih.gov/sra/SRP128487).

### Diversity and statistical analysis

The relative abundance (%) of individual taxa within each community was estimated by comparing the number of sequences assigned to a specific taxon to the number of total sequences obtained for that sample. Alpha diversity analysis, which included the Simpson, Chao1, and Shannon indices, was performed using the summary single command of the MOTHUR software (V.1.31.2, http://www.mothur.org). The community structure was statistically analyzed at different classification levels.

Principal component analysis (PCA) was performed using the R software package (v 2.15.3; https://www.r-project.org/) on the basis of the relative abundance of bacterial and fungal genera. Unweighted pair-group method with arithmetic means (UPGMA) clustering was also performed using QIIME 1.7.0 [27] and displayed using R software (v 2.15.3; http://www.r-project.org). The unweighted UniFrac distance accounts for membership in a community considers both membership and the relative abundance.

Differentially abundant features among the different samples was identified using the linear discriminant analysis (LDA) effect size (LEfSe) pipeline (http://huttenhower.sph.harvard.edu/galaxy/) on the OTU level (relative abundance >1%). Taxa with significant differential abundances were detected by the nonparametric factorial Kruskal–Wallis (KW) rank sum test. The LEfSe analysis was performed under the alpha value for the Kruskal–Wallis test is <0.05, and the threshold on the logarithmic LDA score for discriminative features is >2.0 [28].

Phylogenetic investigation of communities by reconstruction of unobserved states software (PICRUSt, v1.0, http://picrust.github.io/picrust) was used to predict metagenome functional features from 16S rRNA genes [29].

Bacterial and fungal genera in douchi samples were used for network analysis respectively. Spearman’s rank correlations between selected genera were calculated using R package [30]. A valid co-occurrence was selected as a strong correlation if the Spearman’s correlation coefficient (ρ) was greater than 0.6 with a corrected significance level less than 0.01[31–32]. Correlation networks between genera in the samples were constructed and visualized in the Cytoscape software (v 3.2.1; http://www.cytoscape.org) [33].

## Results and discussion

### Sequencing and classification

Total DNA was extracted from the soil samples, after which V4 of the 16S rRNA gene and ITS regions of the 18S rRNA gene were PCR amplified from each DNA sample. PCR products were sequenced using the paired-end method by Illumina Miseq. After quality control, a total of 182,366 high-quality 16S rRNA gene sequences (Table 2) were recovered from the two samples. Additionally, a total of 565,561 validated18S rRNA gene sequences reads were recovered (Table 2). High quality sequences were grouped into 599 OTUs for bacteria and 399 OTUs for fungi, and after removing singletons, the average number of OTUs was 99 for bacteria and 66 for fungi.

**Table 2:** OTUs, Good's Coverage, Chao1, Simpson and Shannon's index for 16S r RNA and 18S r RNA sequencing of the samples.

Sample ID	Reads	OTU	Good's Coverage	Chao1	Shannon
LN1_B	23001	101	98.98%	293.00	2.02
LN2_B	19532	103	98.93%	527.00	4.46
LN3_B	21340	113	99.62%	553.00	4.62
LN_B mean	21291	105	99.18%	457.66	3.70
QY1_B	18529	85	99.39%	799.00	4.92
QY2_B	17642	108	98.89%	680.00	4.56
QY3_B	20003	89	98.81%	671.00	4.26
QY_B mean	18724	94	99.03%	716.66	4.58
LN1_F	109480	82	96.91%	137.00	1.95
LN2_ F	104863	98	97.53%	173.00	2.58
LN3_ F	112394	69	97.62%	112.00	2.12
LN_ F mean	108912	83	96.13%	140.66	2.21
QY1_ F	49246	45	98.39%	68.00	1.64
QY2_ F	100495	47	97.81%	70.00	1.58
QY3_ F	89083	58	97.63%	85.00	1.88
QY_ F mean	79608	50	98.33%	74.33	1.70

**Fig 1:**
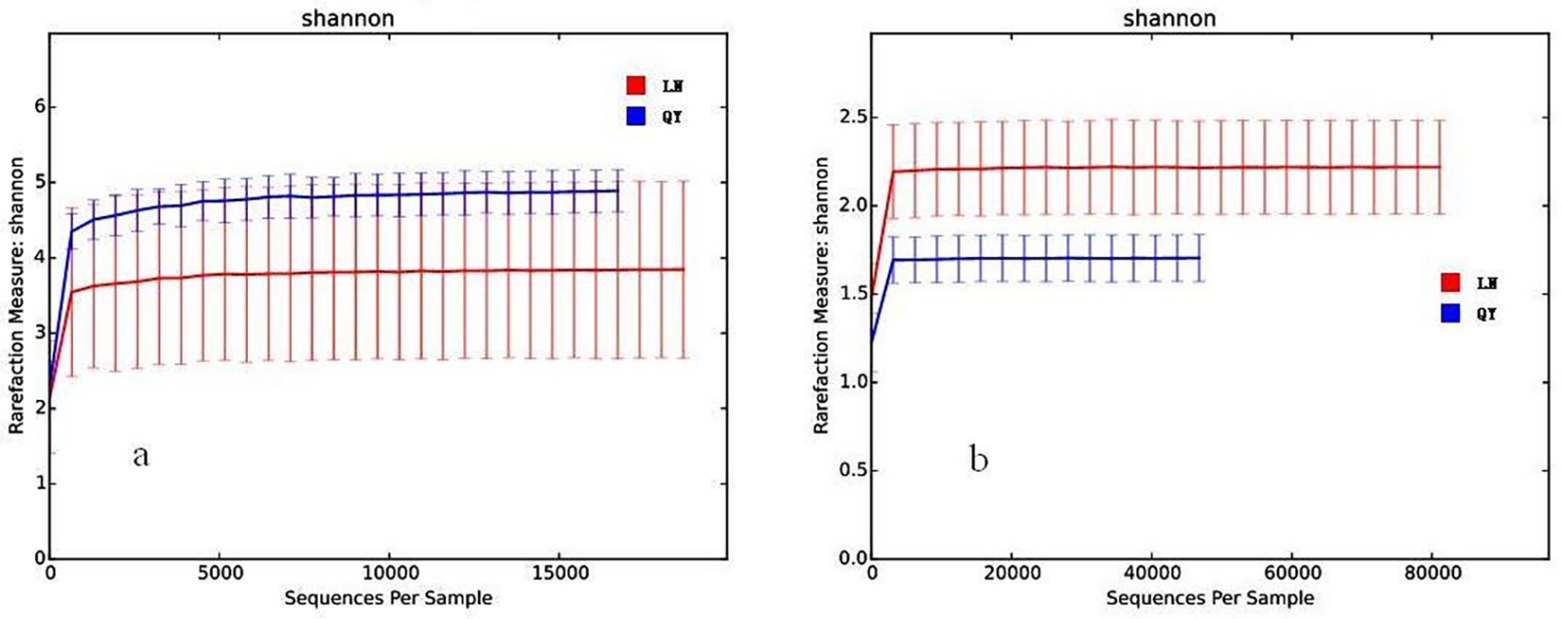
**Rarefaction curves for Shannon diversity indices of bacteria (a) and fungi (b)**.

In the study, the coverage of all the samples ranged from 96.13 to 99.62%, which indicated an adequate level of sequencing to identify most diversity in the samples. In addition, rarefaction curves for Shannon diversity indices for bacteria and fungi are shown in Fig 1A and 1B, respectively. The rarefaction curves were nearly parallel with the x-axis and the Shannon diversity index reached saturation, suggesting that although new phylotypes would be expected with additional sequencing, the majority of bacterial and fungal phylotypes present in douchi had been captured already.

### Analysis of alpha diversity

Two indices were determined (Chao1 richness index and Shannon index) to measure the alpha diversity of the microbiome in the analyzed sample (Table 2). The microbial diversity was compared, as estimated by the Shannon index, and the results showed that the index of bacteria was significantly higher than that of fungi in all the samples (P < 0.05). The result also showed that the bacterial diversity difference was not statistically significant (P > 0.05) in douchi collected in Longnan and Qinyang. However, the fungal diversity was significantly different (P < 0.05) in samples from different origins, indicating that geography may have impacted the fungal diversity in the samples.

We also compared the microbial species richness, as estimated by the Chao1 richness index, and the results showed that the index of bacteria was significantly higher than that of fungi in all the samples (P<0.05). The result also showed that bacterial and fungal species richness was significantly different (P < 0.05) in samples from different origins, indicating that geography may have impacted the microbial species richness in the samples. The same results were found for the fermented milk matsoni and tarag [20, 34].

### Comparison of bacterial communities in the samples

**Fig 2:**
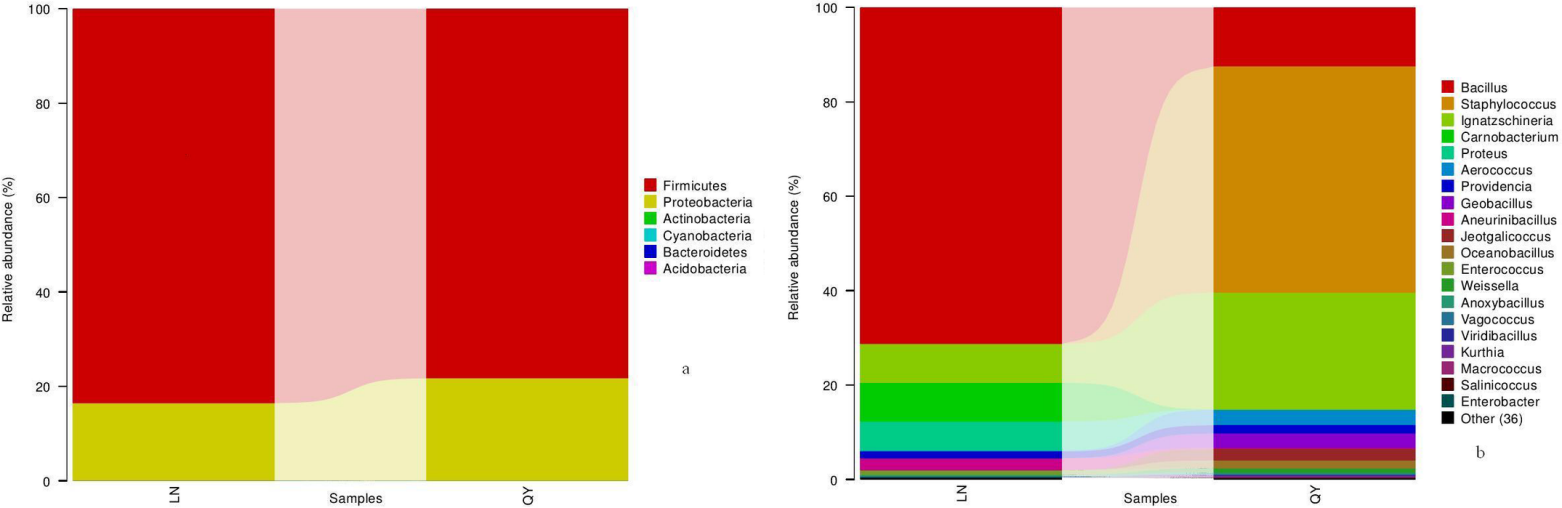
**Principal components analysisfor bacteria (a) and fungi (b)**.

We examined the composition of the bacterial community in the samples. A total of six phyla were identified in the two samples via taxonomic summary, with Firmicutes and Proteobacteria being dominant and having a relative abundance >5% (Fig 2A). The relative abundance of Firmicutes, the most abundant phyla in the samples, ranged from 71.5% to 93.2%. Proteobacteria was the second most abundant phyla in the samples, with a relative abundance of 6.7–28.4%. The relative abundance difference of the above two dominant phyla was not significant between samples from Qingyang and Longnan (P>0.05). The relative abundance of the other four phyla was lower than 1% and differed between the samples from Qingyang and Longnan. Firmicutes, Proteobacteria, Actinobacteria, and Bacteroidetes were also observed in previous studies, while Acidobacteria and Cyanobacteria were not detected in previous studies [1, 3, 8,18].

A total of 56 bacterial genera were identified in the samples via taxonomic summary. The abundance of the top 20 bacterial genera in the samples is shown in Fig 2B. Two dominant genera (*Bacillus* and *Ignatzschineria*) were shared by the two samples, both of which had a relative abundance higher than 1%. Additionally, four genera (*Staphylococcus*, *Aerococcus*, *Geobacillus*, and *Jeotgalicoccus*) were only dominant in the sample from Qingyang. Among them, *Staphylococcus* was the most-dominant genus in the samples from Qingyang, with a relative abundance of 38.3±6.0%. Four genera (*Carnobacterium*, *Proteus*, *Aneurinibacillus*, and *Enterococcus*) were only dominant in the sample from Longnan. Among these, *Carnobacterium* was the third most abundant genus in the samples from Longnan, with a relative abundance of 8.2±6.5%. Specifically, the abundance of *Bacillus* in the samples from Qingyang and Longnan was 10.2±3.9% and 69.2±17.9%, which was the largest difference among genera. Most of the bacterial genera detected in the present study, including *Bacillus*, *Ignatzschineria*, *Providencia*, *Staphylococcus*, *Weissella*, *Acinetobacter*, *Lactobacillus*, *Enterobacter* and *Pseudomonas*, were observed in previous studies [1, 3, 8,18]. In previous studies, some species of bacterial genera such as *Bacillus*, *Lactococcus*, *Lactobacillus*, *Enterococcus*, and *Weissella* have probiotic potential [1, 3,35]. However, members of *Staphylococcus*, *Enterobacter* and *Aerococcus* have been involved in human infections as opportunistic pathogens [1, 3]. The results showed that the home-made douchi prepared in a traditional way might not be safe, and that it should be heated when serving.

**Fig 3:**
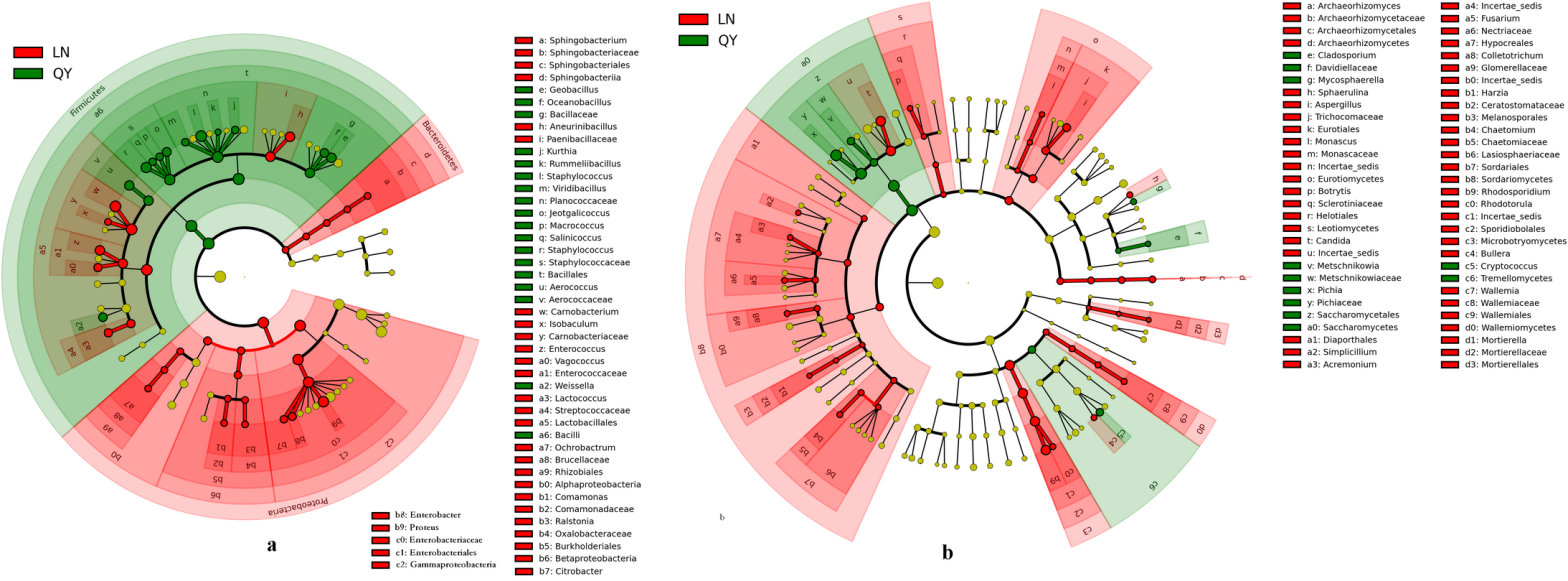
**Relative abundance of bacterial phyla (a) and genera (b) in samples**.

Based on linear discriminant analysis (LDA), we also found a significant difference in bacterial community compositions in douchi from Qingyang compared with Longnan (Fig 3A). We observed a total of 52 OTUs, with 19 OTUs being found in samples from Longnan and 33 in samples from Qingyang, which was significantly different.

### Comparison of fungal communities in the samples

**Fig 4:**
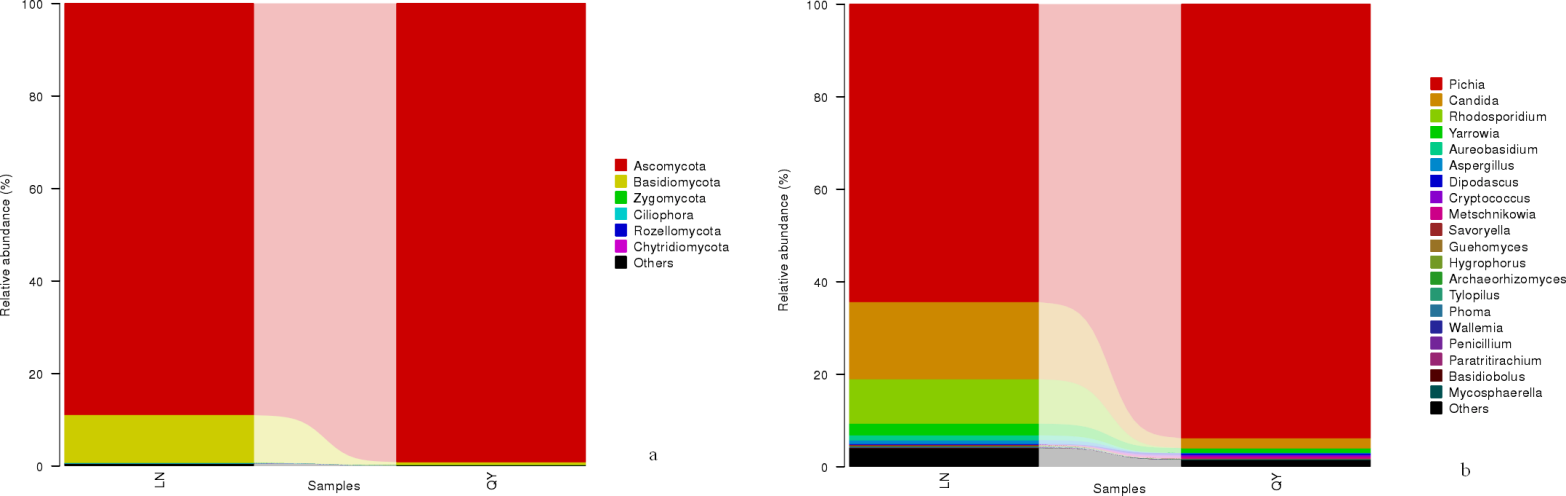
**Linear discriminant analysis of microbial community compositions in douchi samples for bacteria (a) and fungi (b)**. The node size represents the difference in relative abundance. Green or red nodes indicate OTUs with significant differences of relative abundance, yellow nodes indicate OTUs with no significant differences in relative abundance.

Fig 4A shows the difference in fungal abundance among samples at phylum level. A total of six phyla were identified in the two samples via taxonomic summary. The relative abundance of Ascomycota, the most dominant phyla, was 89.5±16.0% and 99.3±0.3% in samples from Longnan and Qingyang. Basidiomycota was dominant only in the sample from Longnan, with a relative abundance of 9.8±5.6%. Levels of another four phyla present with a relative abundance lower than 1% differed between samples from Qingyang and Longnan. Ascomycota, Basidiomycota, and Zygomycota were observed in previous studies and the present study, while Ciliophora, Rozellomycota, and Chytridiomycota were observed in previous studies, but they were not detected in the present study [1, 3, 8,18].

The abundance of the top 20 fungal genera in the samples is shown in Fig 4B. Two dominant genera (*Pichia* and *Candida*) were shared by the two samples, both of which had a relative abundance higher than 1%. Additionally, two genera (*Rhodosporidium* and *Yarrowia*) were dominant only in the sample from Longnan. Among them, *Pichia* was the most-dominant genus in the samples from Longnan and Qingyang, with relative abundances of 64.5±7.3% and 93.8±0.2%, respectively. *Candida* was the second most abundant genus in samples from Longnan and Qingyang, with relative abundances of 16.9±8.6% and 2.4±1.5%, respectively. Some of the fungal genera detected in the present study, including *Monascus*, *Fusariums*, *Penicillium*, and *Aspergillus*, were observed in previously investigated fungal douchi samples [1, 3, 8, 18], while many of the genera detected in the previous studies, such as *Mucor*, *Geotrichum*, *Scopulariopsi*, *Rhizopus*, *Trichothecium*, and *Lichtheimia*, were not detected in the present study [1, 3, 8, 18]. In the fungal genera, some species of *Pichia* and *Aspergillus* were reported as probiotics in Douchi samples [1, 3]. As for *Candida*, a genus of yeast, always appears as large, round, white or cream colonies on agar plates [36, 37]. Many species of this genus are responsible for candidemia [38]. Other species, such as *C*.*oleophila* have been used as biological agents to prevent the growth of the bacteria pathogen [39].

Based on linear discriminant analysis (LDA), a significant difference was found in fungal community compositions in douchi from Qingyang compared with Longnan (Fig 3B). Specifically, the levels of OTUs differed significantly among groups, with 53 OTUs being observed in samples from Longnan and 11 in samples from Qingyang.

### Analysis of beta diversity

**Fig 5:**
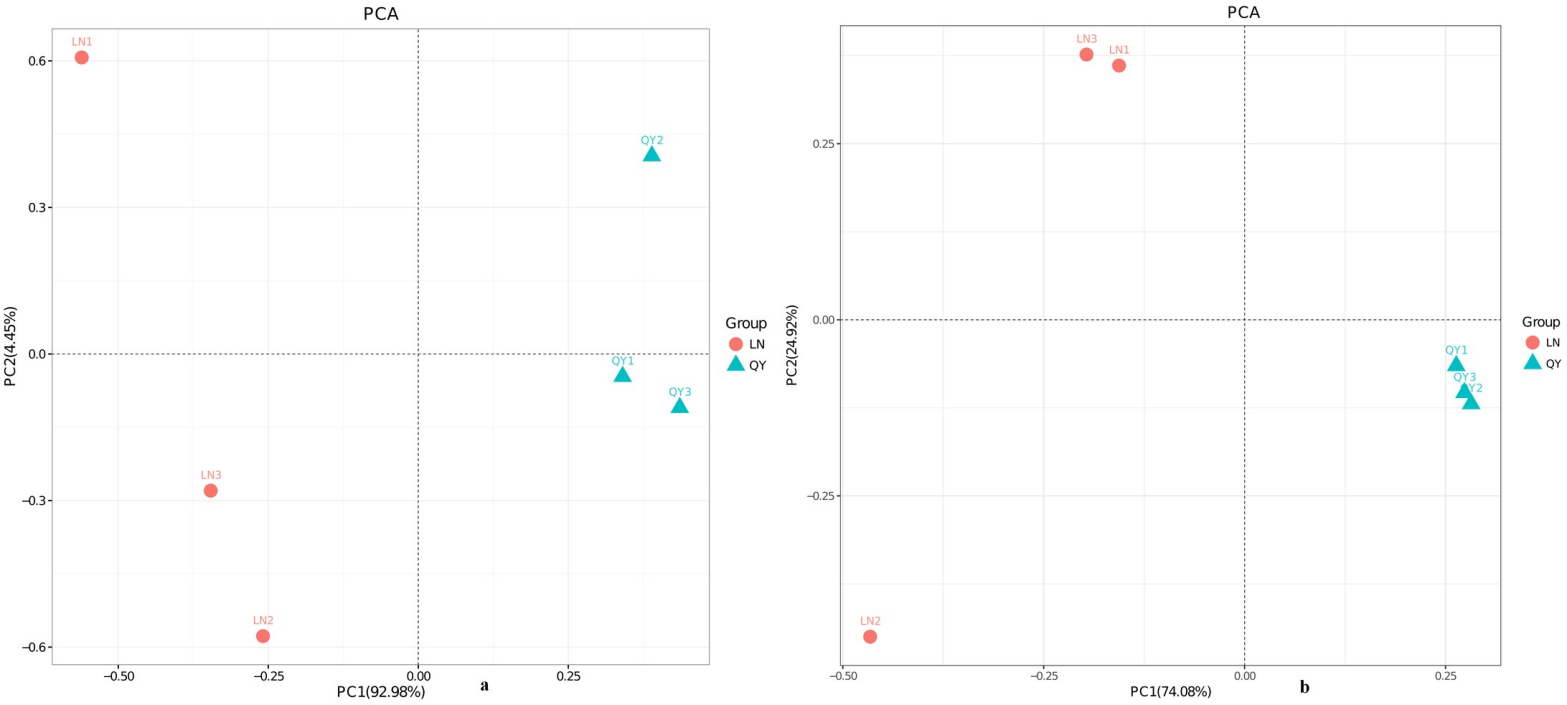
**Relative abundance of fungal phyla (a) and genera (b) in the samples**.

**Fig 6:**
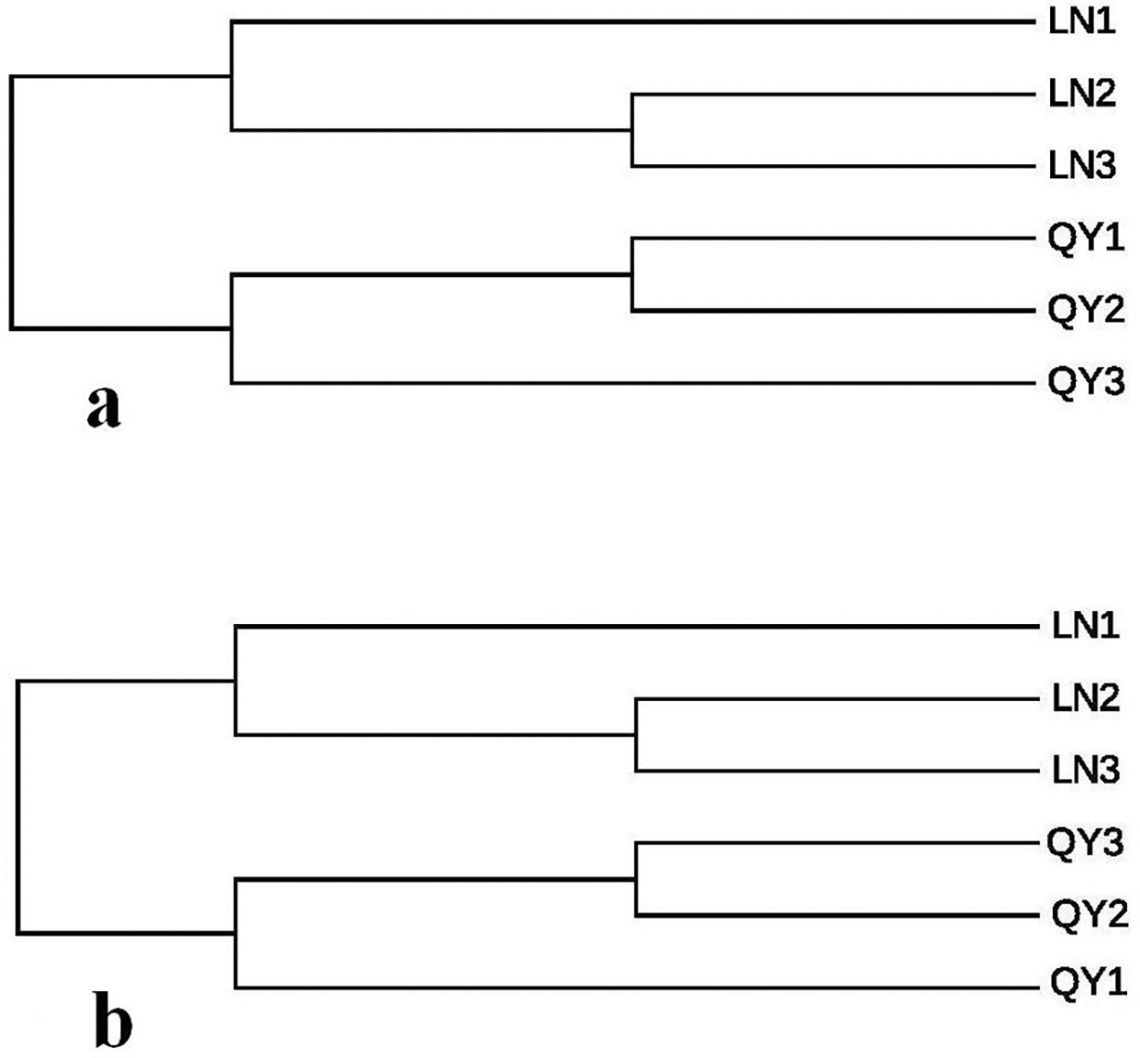
**Unweighted pair-group analysis (UPGMA) of bacteria (a) and fungi (b) using arithmetic means**.

**Fig 7:**
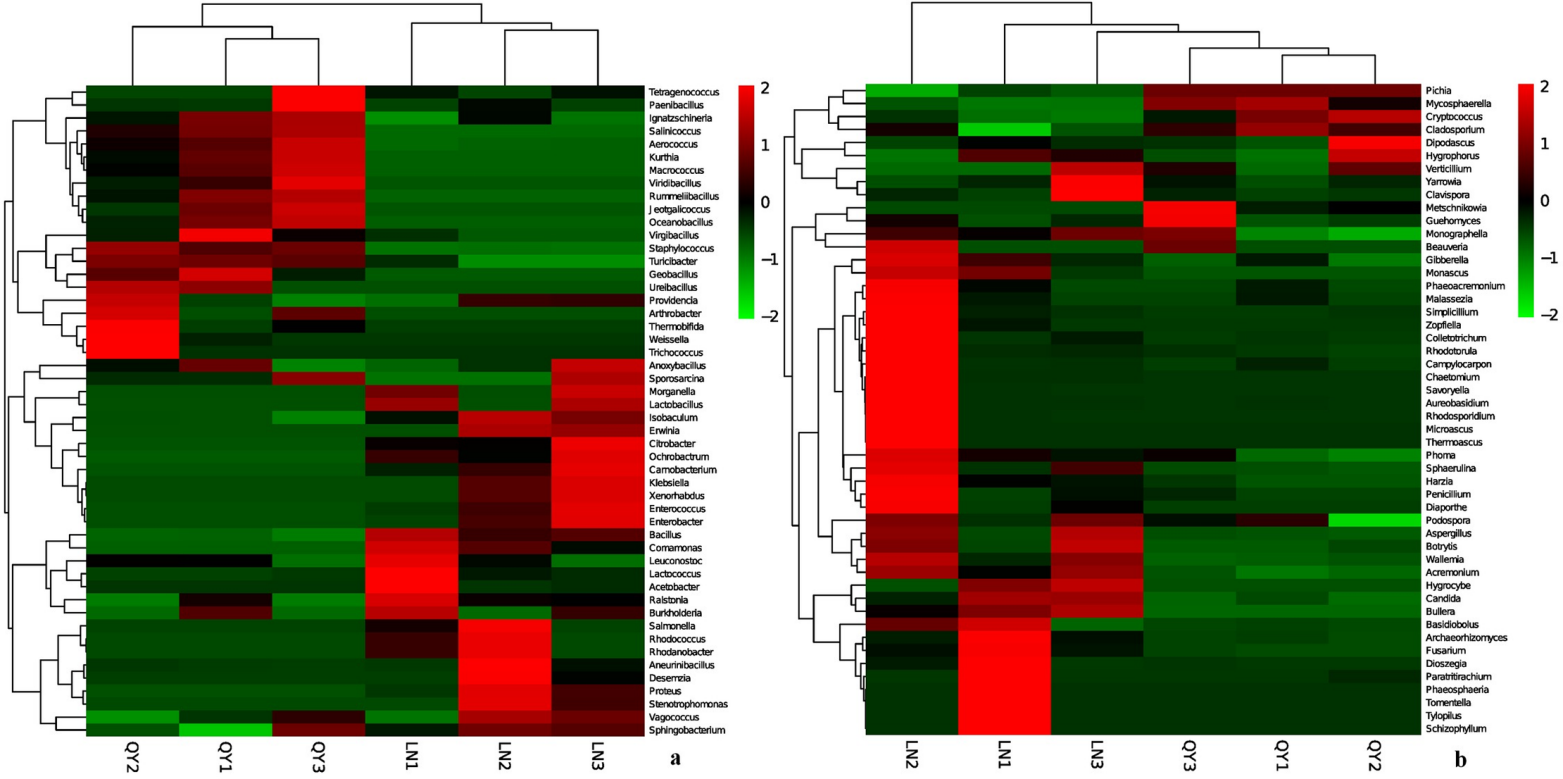
**Heatmap and dendrogram of abundant bacterial (a) and fungal (b) genera present in the samples**. Color blocks represent the relative abundance of genera, namely the Z value. More green indicates a higher relative abundance.

Principal component analysis (PC1 = 92.98%, PC2 = 4.45%; Fig 5A) based on the relative abundance of bacterial community showed that samples from the same location were not closely grouped together (Fig 5A).Geographic variations were much clearer when comparing samples from different regions using unweighted pair-group analysis (UPGMA), highlighting the production region as a salient factor affecting the bacterial composition of the different samples (Fig 6A). A heatmap (Fig 7A) at the top 50 bacterial genus level revealed that samples of the same origin cluster together, showing a high degree of similarity between samples of the same origin and a low degree of similarity among samples of different origin.

Principal component analysis (PC1 = 74.08%, PC2 = 24.92%; Fig 5B) for fungal microbiota demonstrated that samples from Longnan and Qingyang were well separated and samples from the same location were closely grouped together except for LN2 (Fig 5B).

UPGMA based on fungal microbiota communities revealed a distinct clustering pattern between samples of different origin (Fig 6B). Similarly, heatmap analysis also indicated a significant difference in fungal genera between samples of different origin (Fig 7B).

Thus, it can be concluded that the microbial flora in different samples was stratified by geographic region. The douchi samples used in this study were produced by similar traditional preservation methods. Therefore, it is likely that the differences in microbial structure between samples of different origin can be attributed to geographic location and other environmental factors.

### Functional genes of the bacteria present in douchi samples

The PICRUSt software was used to predict the functional genes of the bacteria present in samples and their metabolic pathways [40–42]. Of the functional microbial genes in the samples, 45.72–47.99% were related to the following metabolic pathways: AA metabolism, carbohydrate metabolism, energy metabolism, metabolism of cofactors and vitamins, nucleotide metabolism, lipid metabolism, xenobiotics biodegradation and metabolism, enzyme families, metabolism of terpenoids and polyketides, metabolism of other AA, glycanbio synthesis and metabolism, and biosynthesis of other secondary metabolites (Table 3). Genes involved in AA metabolism and carbohydrate metabolism predominated in samples from Qingyang and Longnan, accounting for 20.12% and 19.83% of all genes, respectively. Other functional genes of the bacteria present in samples were related to cellular processes, environmental information processing, genetic information processing, human diseases, organismal systems and others (Table 4). Among these, genes involved in membrane transport were dominant in samples from Qingyang and Longnan, accounting for 12.00% and 12.82% of all genes, respectively.

**Table 3:** The functional features relating to metabolism of genes from bacteria in the samples.

Metabolic pathway	Percentage composition in samples							
	LN1	LN2	LN3	LN_mean_	QY1	QY2	QY3	QY_mean_
Amino Acid Metabolism	10.33%	9.66%	9.42%	9.80%	10.04%	9.96%	10.01%	10.00%
Biosynthesis of Other Secondary Metabolites	0.61%	0.68%	0.64%	0.64%	0.62%	0.55%	0.62%	0.60%
Carbohydrate Metabolism	10.22%	9.61%	10.27%	10.03%	9.94%	10.50%	9.93%	10.12%
Energy Metabolism	5.37%	5.18%	4.99%	5.18%	5.54%	5.55%	5.56%	5.55%
Enzyme Families	1.89%	1.93%	1.99%	1.94%	1.80%	1.83%	1.78%	1.80%
Glycan Biosynthesis and Metabolism	1.12%	1.65%	1.53%	1.43%	1.66%	1.62%	1.69%	1.66%
Lipid Metabolism	3.69%	3.52%	3.50%	3.57%	3.62%	3.63%	3.61%	3.62%
Metabolism of Cofactors and Vitamins	4.49%	4.11%	3.83%	4.14%	4.40%	4.47%	4.39%	4.42%
Metabolism of Other Amino Acids	1.84%	1.84%	1.76%	1.81%	1.83%	1.78%	1.82%	1.81%
Metabolism of Terpenoids and Polyketides	1.85%	1.78%	1.85%	1.83%	1.79%	1.79%	1.79%	1.79%
Nucleotide Metabolism	3.38%	3.45%	3.57%	3.47%	3.70%	3.84%	3.74%	3.76%
Xenobiotics Biodegradation and Metabolism	2.57%	2.31%	2.56%	2.48%	2.40%	2.47%	2.39%	2.42%

**Table 4:** Other functional genes from bacteria in the samples.

Function	Percentage composition in samples							
	LN1	LN2	LN3	LN_mean_	QY1	QY2	QY3	QY_mean_
Cell Growth and Death	0.40%	0.41%	0.40%	0.40%	0.48%	0.49%	0.48%	0.48%
Cell Motility	4.25%	4.28%	3.84%	4.12%	3.04%	2.22%	2.93%	2.73%
Transport and Catabolism	0.28%	0.24%	0.23%	0.25%	0.23%	0.23%	0.23%	0.23%
Membrane Transport	12.29%	12.67%	13.49%	12.82%	11.91%	12.18%	11.91%	12.00%
Signaling Molecules and Interaction	0.17%	0.18%	0.18%	0.18%	0.17%	0.16%	0.17%	0.17%
Signal Transduction	2.06%	2.36%	2.20%	2.21%	2.19%	2.09%	2.18%	2.15%
Folding, Sorting and Degradation	2.10%	2.35%	2.22%	2.22%	2.61%	2.55%	2.65%	2.60%
Replication and Repair	7.43%	7.57%	7.69%	7.56%	8.10%	8.12%	8.18%	8.13%
Transcription	3.08%	2.81%	2.95%	2.95%	2.46%	2.53%	2.42%	2.47%
Translation	4.42%	4.55%	4.60%	4.52%	5.15%	5.18%	5.22%	5.18%
Cancers	0.08%	0.11%	0.09%	0.09%	0.11%	0.10%	0.11%	0.11%
Immune System Diseases	0.04%	0.05%	0.05%	0.05%	0.05%	0.05%	0.06%	0.05%
Infectious Diseases	0.33%	0.44%	0.40%	0.39%	0.48%	0.54%	0.49%	0.50%
Metabolic	0.07%	0.07%	0.07%	0.07%	0.08%	0.08%	0.08%	0.08%
Neurodegenerative	0.28%	0.29%	0.24%	0.27%	0.31%	0.27%	0.32%	0.30%
Circulatory System	0.01%	0.03%	0.02%	0.02%	0.05%	0.03%	0.05%	0.04%
Digestive System	0.06%	0.04%	0.05%	0.05%	0.04%	0.04%	0.04%	0.04%
Endocrine System	0.34%	0.29%	0.27%	0.30%	0.25%	0.22%	0.25%	0.24%
Environmental Adaptation	0.14%	0.15%	0.15%	0.15%	0.13%	0.12%	0.13%	0.13%
Excretory System	0.03%	0.02%	0.02%	0.02%	0.00%	0.00%	0.00%	0.00%
Immune System	0.01%	0.04%	0.04%	0.03%	0.04%	0.03%	0.04%	0.04%
Nervous System	0.07%	0.08%	0.06%	0.07%	0.09%	0.08%	0.09%	0.09%

**Fig 8:**
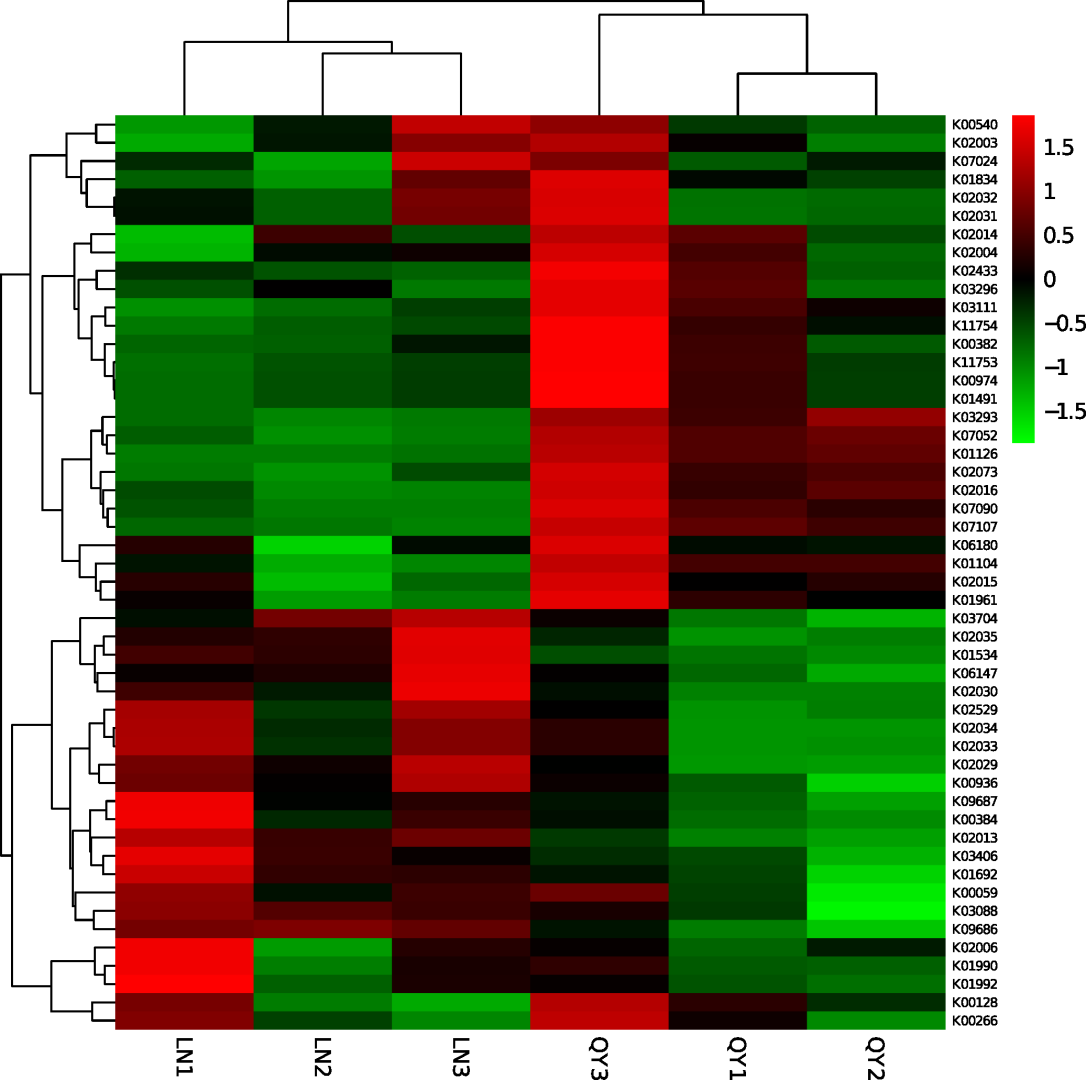
Heatmap of clustering analysis based on the top 50 functional genes in the samples.

A heatmap (Fig 8) was plotted to estimate the similarities of the samples at the top 50 functional gene level. The results indicated that the highest degree of similarity was among samples of the same origin and that a significant difference was observed between bacterial communities of samples of different origin. These results were consistent with our previous cluster analysis results of the bacterial community (Fig 7A).

### Microbial interactions in the samples

**Fig 9:**
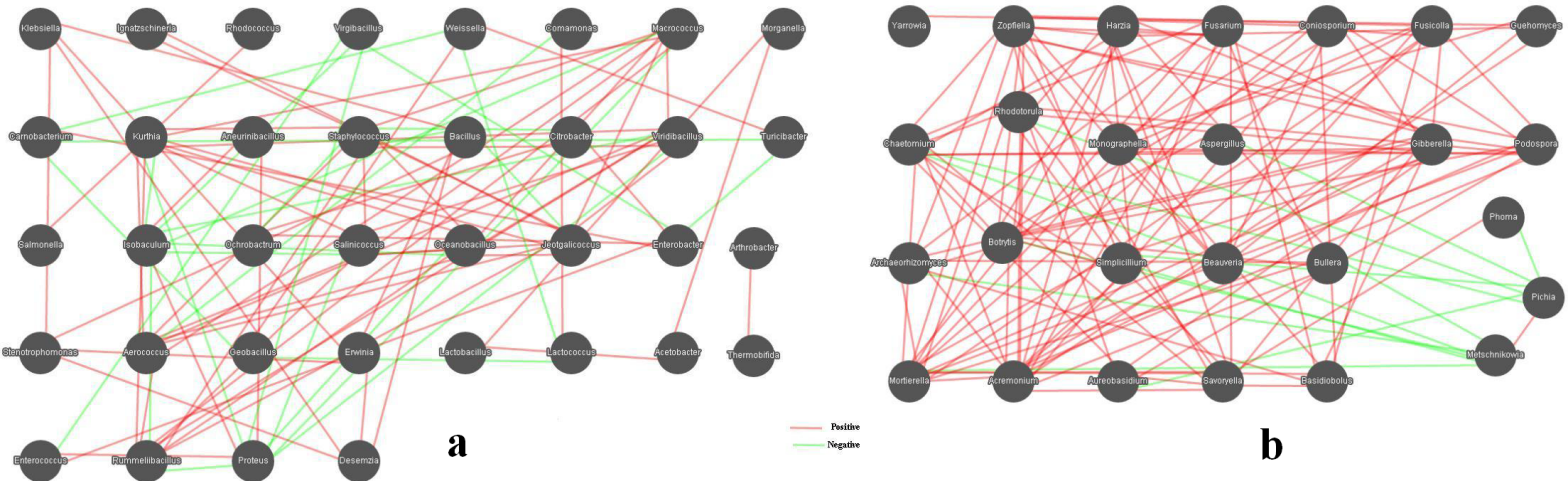
**Networks of microbial interaction for bacteria (a) and fungi (b) in the samples**. Each node represents a bacterial or fungal genus, edges denote significant correlations between phylotypes (Spearman’s ρ> 0.6). Positive correlations colored in green indicate co-occurrence, whereas negative correlations in red indicate mutual exclusion.

Microorganisms do not exist in isolation, but instead form complex, interacting ecological webs [43–45]. Microbial networks have been inferred for a range of communities, from soil and ocean communities to human body communities [46–49]. In the present study, relationships between genera in the douchi samples were calculated using the Spearman rank correlation coefficient and visualized as a network with the Cytoscape software (Fig 9A and 9B).

The network for bacterial community in samples consists of 36 nodes and 92 edges (Fig 9A). Obviously, the network is dominantly cooperative, and the ratio of cooperative vs. non-cooperative interactions is 65:27. *Bacillus*, *Staphylococcus*, *Jeotgalicoccus*, *Oceanobacillus*, *Viridibacillus*, *Kurthia*, *Salinicoccus*, *Rummeliibacillus*, *Isobaculum*, *Aerococcus*, *Macrococcus*, *Proteus*, and *Enterobacter* were found to be hub genera (≥ 6 edges per node) in the network (Fig 9A). *Bacillus*, the dominant genera, had a negative impact on the other two dominant genera (*Staphylococcus* and *Jeotgalicoccus*), while it was positively correlated with four non-dominant genera (*Klebsiella*, *Erwinia*, *Desemzia*, *Stenotrophomonas*). *Ignatzschineria*, the second most abundant genera, was positively associated with two other dominant genera (*Staphylococcus* and *Jeotgalicoccus*). Aditionally, *Turicibacter* have a positive impact on *Thermobifida*, while it had no relationship with other genera.

A total of 20 nodes (genera) and 23 edges (pairs of significant and robust correlations) were found in the network for fungal community in samples (Fig 9B). In the network for fungi, the ratio of cooperative vs. non-cooperative interactions is 34:12. As shown in Fig 9B, *Pichia*, *Rhodotorula*, *Chaetomium*, *Acremonium*, *Aspergillus*, and *Sphaerulina* comprised hub genera (≥ 3 edges per node) in the fungal network. *Pichia*, the dominant genera, had a negative impact on the other four genera. *Sphaerulina*, the non-dominant genera, was negatively correlated with five other non-dominant genera, while it was negatively correlated with *Pichia*. Aditionally, *Candida*, the second-dominant genera shared by the samples, have a positive impact on *Archaeorhizomyces*, while they shared no relationship with other genera. Overall, the results indicated that microbial interactions between bacterial communities and fungal communities in the samples were very complex.

## Conclusions

Metagenomics has been shown to be a powerful tool in exploring a large diversity of natural environments; however, few studies have considered food microbiota until recently. In this study, microbial diversity and communities in two kinds of douchi from Gansu province in northwest China were studied by high-throughput sequencing. This is the first study to apply this technology to study food ecology and microbial interactions in bacterial douchi. The results presented herein provide insights into the impact of environments on microbial communities in douchi and lay a foundation for further investigations into the food ecology of douchi.

## Supporting information

S1 File Raw data for 16s rRNA in samples. (RAR)

S2 File Raw data for ITS rRNA in samples. (RAR)
