# Correction: Microbial diversity in two traditional bacterial douchi from Gansu province in northwest China using Illumina sequencing

**DOI:** 10.1371/journal.pone.0197527

**Published:** 2018-05-10

**Authors:** Weibing Zhang, Qiaoqiao Luo, Yan Zhu, Jiang Ma, Lei Cao, Min Yang, Pencheng Wen, Zhongmin Zhang, Xiaoling He

The figure legends for Fig 2, Fig 3, Fig 4 and Fig 5 are incorrect. Please see the corrected figures here.

**Fig 2:**
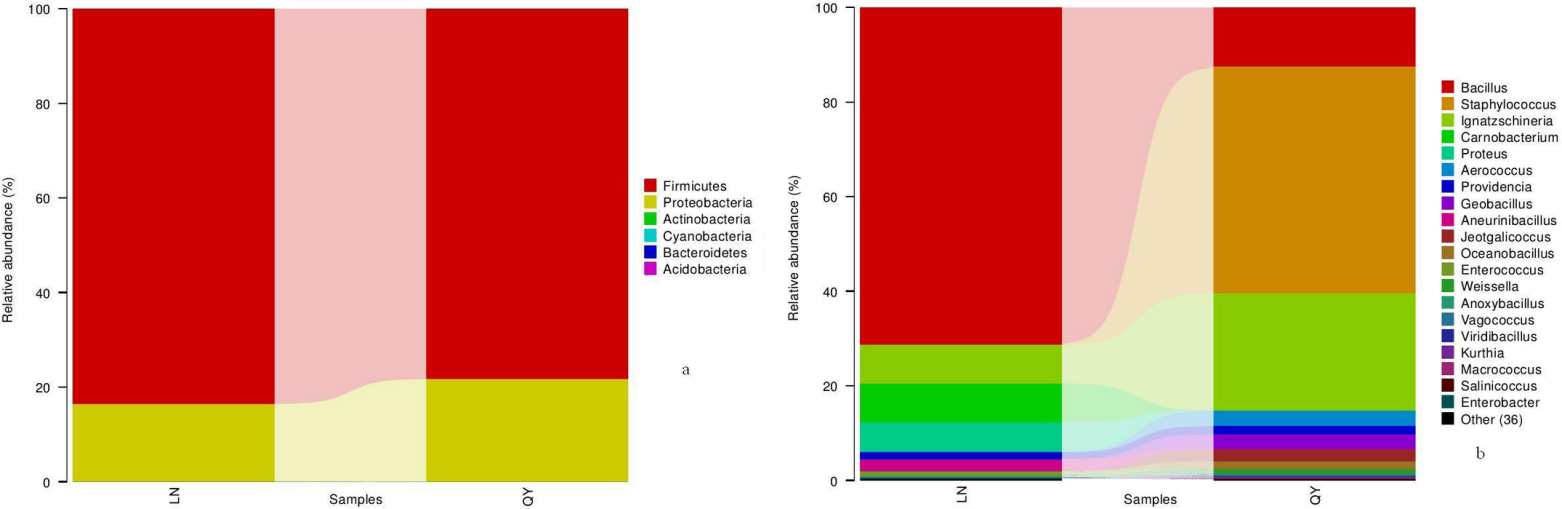
**Relative abundance of bacterial phyla (a) and genera (b) in samples**.

**Fig 3:**
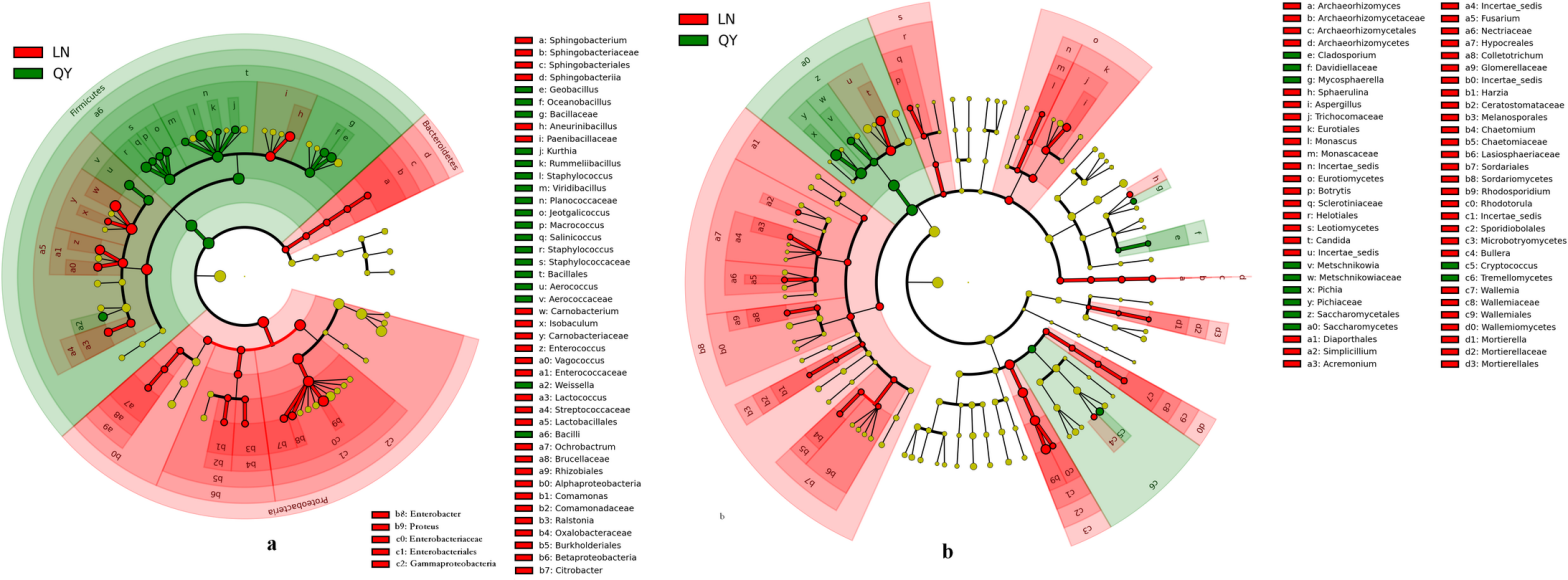
**Linear discriminant analysis of microbial community compositions in douchi samples for bacteria (a) and fungi (b).** The node size represents the difference in relative abundance. Green or red nodes indicate OTUs with significant differences of relative abundance, yellow nodes indicate OTUs with no significant differences in relative abundance.

**Fig 4:**
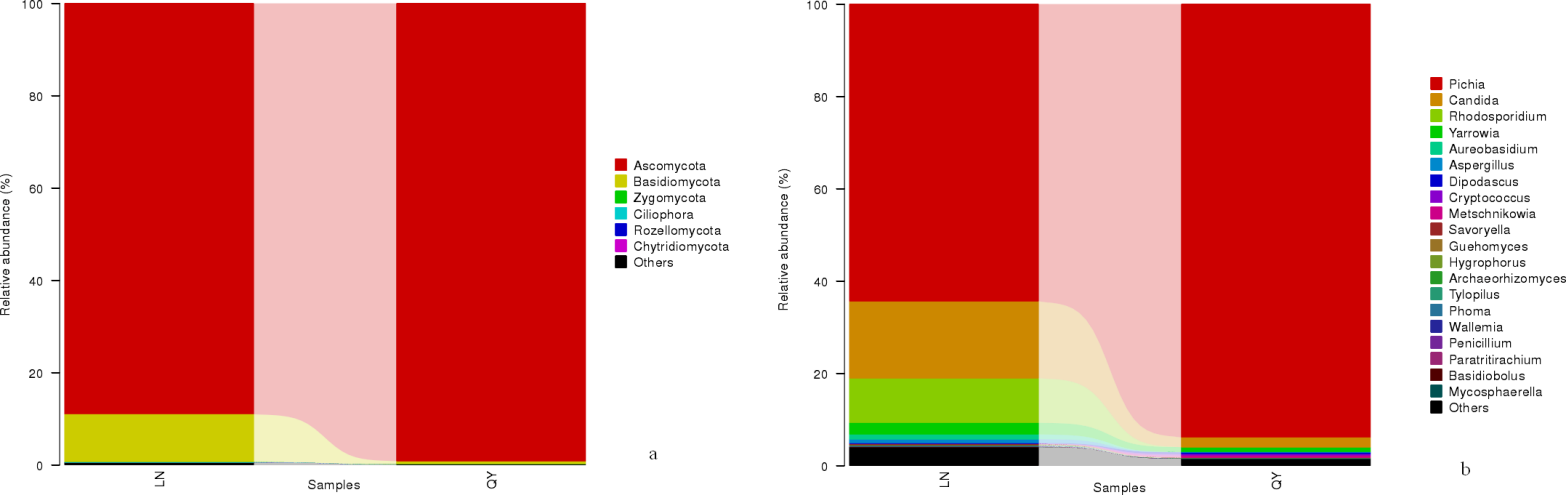
**Relative abundance of fungal phyla (a) and genera (b) in the samples**.

**Fig 5:**
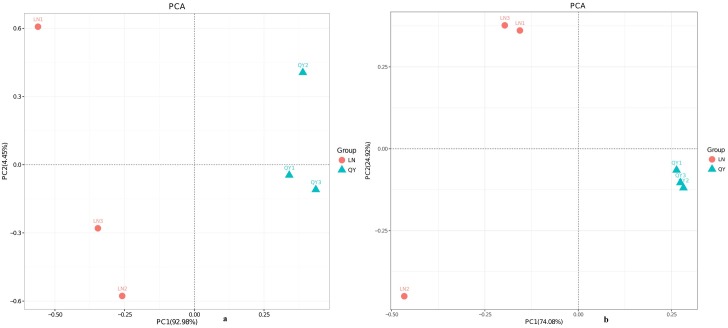
**Principal components analysis for bacteria (a) and fungi (b)**.
